# Ferroelectric Orthorhombic ZrO_2_ Thin Films Achieved Through Nanosecond Laser Annealing

**DOI:** 10.1002/advs.202207390

**Published:** 2023-03-22

**Authors:** Anna P. S. Crema, Marian C. Istrate, Alexandre Silva, Veniero Lenzi, Leonardo Domingues, Megan O. Hill, Valentin S. Teodorescu, Corneliu Ghica, Maria J. M. Gomes, Mario Pereira, Luís Marques, Judith L. MacManus‐Driscoll, José P. B. Silva

**Affiliations:** ^1^ Physics Center of Minho and Porto Universities (CF‐UM‐UP) University of Minho Campus de Gualtar Braga 4710‐057 Portugal; ^2^ Laboratory of Physics for Materials and Emergent Technologies LapMET University of Minho Braga 4710‐057 Portugal; ^3^ University of Bucharest Faculty of Physics Atomistilor 405, Magurele Ilfov 077125 Romania; ^4^ National Institute of Materials Physics Lab. of Atomic Structures and Defects in Advanced Materials 405A Atomistilor Str. Magurele Ilfov 077125 Romania; ^5^ Dept. of Materials Science and Metallurgy University of Cambridge 27 Charles Babbage Rd Cambridge CB3 OFS United Kingdom

**Keywords:** binary oxides, ferroelectricity, nanosecond laser annealing, orthorhombic phase

## Abstract

A new approach for the stabilization of the ferroelectric orthorhombic ZrO_2_ films is demonstrated through nanosecond laser annealing (NLA) of as‐deposited Si/SiO*_x_*/W(14 nm)/ZrO_2_(8 nm)/W(22 nm), grown by ion beam sputtering at low temperatures. The NLA process optimization is guided by COMSOL multiphysics simulations. The films annealed under the optimized conditions reveal the presence of the orthorhombic phase, as confirmed by X‐ray diffraction, electron backscatter diffraction, and transmission electron microscopy. Macroscopic polarization‐electric field hysteresis loops show ferroelectric behavior, with saturation polarization of 12.8 µC cm^−2^, remnant polarization of 12.7 µC cm^−2^ and coercive field of 1.2 MV cm^−1^. The films exhibit a wake‐up effect that is attributed to the migration of point defects, such as oxygen vacancies, and/or a transition from nonferroelectric (monoclinic and tetragonal phase) to the ferroelectric orthorhombic phase. The capacitors demonstrate a stable polarization with an endurance of 6.0 × 10^5^ cycles, demonstrating the potential of the NLA process for the fabrication of ferroelectric memory devices with high polarization, low coercive field, and high cycling stability.

## Graphical abstract

Nanosecond laser annealing of amorphous ZrO_2_ thin films is shown to produce the orthorhombic structure with ferroelectric properties: saturation polarization of 12.8 µC cm^−2^, remnant polarization of 12.7 µC cm^−2^ and coercive field of 1.2 MV cm^−1^. Thus, the creation of ZrO_2_‐based memory devices by a simple industrial process is demonstrated.

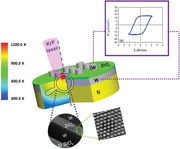

## 1 Introduction

Ferroelectrics research field has rejuvenated in the last decade due to the discovery of ferroelectricity in simple binary or ternary fluorite‐based oxide thin films.^[^ ^1^ ^]^

The interest in these materials arises from their compatibility with complementary metal‐oxide semiconductors (CMOS) where high‐temperature‐grown perovskites present more challenges. Hence, compared to conventional perovskites, they have a lower temperature of fabrication as well as high scalability potential. Thus, these fluorite‐based films could solve the energy efficiency problem of scaled semiconductors in a number of ways, e.g. when used in ferroelectric capacitors, transistors or tunnel junctions, negative capacitance devices, logic‐in‐memory, neuromorphic computing. Other energy storage, pyroelectric or piezoelectric‐based applications are also possible.^[^ ^1^, ^2^ ^]^

In 2011, Böscke et al. reported for the first time on the ferroelectric properties of Si‐doped HfO_2_ thin films.^[^ ^3^ ^]^ Many research groups followed this path and investigated several other dopants such as La, Gd, Y, Al, etc, either in polycrystalline or epitaxial films.^[^ ^4^, ^5^, ^6^, ^7^ ^]^

Due to the possibility of achieving a wide range of properties ranging from dielectric to ferroelectric and antiferroelectric, Hf*_x_* Zr_1‐_ *_x_* O_2_ (HZO), with *x* from 0 up to 1, is a very promising composition compared to other doped HfO_2_ compositions because it gives the possibility of using these materials in nonvolatile memory devices and energy storage capacitors.^[^ ^1^, ^8^, ^9^ ^]^ Dopant concentration, strain, electrode materials, and the upper capping layer are all reported to influence the polymorphism in HZO films.^[^ ^10^, ^11^, ^12^ ^]^ One of the most promising strategies used to stabilize the orthorhombic (*o‐*) phase and enhance the ferroelectric properties of HZO films was the use of tensile stress.^[^ ^13^, ^14^, ^15^ ^]^ Different tensile stress levels can be obtained by the appropriate choice of the electrodes, since the difference in the thermal expansion coefficient (TEC) of HZO and the electrode is one of the important origins of tensile stress when the films are cooled down to room temperature.

Different electrode materials such as TiN,^[^ ^16^, ^17^, ^18^ ^]^ Pt,^[^ ^17^ ^]^ W,^[^ ^17^ ^]^ La_0.67_Sr_0.33_MnO_3_^6^, Pb_2_Ir_2_O_7_,^[^ ^18^ ^]^ etc. have been investigated. Among them, TiN is widely used as an electrode to induce ferroelectricity in HZO‐based capacitors. However, it has a high TEC (9.1 × 10^−6^ K^−1^) comparable with that of HZO film (10 × 10^−6^ K^−1^),^[^ ^15^ ^]^ which is not beneficial for the suppression of nonferroelectric (i.e., *c*, *t*, and *m*) phases, resulting in relatively low ferroelectric polarization. In this context, tungsten (W) emerges as the best choice since it exhibits the lowest TEC (4.5 × 10^−6^ K^−1^)^[^ ^19^ ^]^ that allows to achieve high polarization values. However, *W* is a reactive metal that could easily react with oxygen in the HZO layer, leading to the formation of WO*_x_* layer that can degrade the device performance.^[^ ^20^ ^]^

Although HZO shows the largest polarisation values, ZrO_2_ is much more abundant in nature and therefore is a more appealing choice for large‐scale use.^[^ ^21^ ^]^ The emergence of the ferroelectricity in ZrO_2_ arises from the *o‐*phase (space group: Pca2_1_)^[^ ^22^ ^]^ and the polar rhombohedral *(r‐)*phase (space group: R3m)^[^ ^23^ ^]^ that can be only achieved in a constrained environment in the thin film form. It was recently revealed that ferroelectricity and hysteretic polarization switching can be achieved in ultrathin ZrO_2_ with the fluorite‐structure unit‐cell size^[^ ^24^ ^]^ for a film thickness of 5 Å. However, pure ZrO_2_ films have shown a moderate polarization,^[^ ^23^ ^]^ in spite of the theoretical predictions that point to a high polarization value close to the one observed in doped HfO_2_.^[^ ^25^ ^]^ Therefore, ZrO_2_ thin films require further optimization. However, to the best of our knowledge the potential use of W electrodes to fabricate ferroelectric devices based on ZrO_2_ thin films was not yet investigated in spite of the TEC coefficient of ZrO_2_ (10.5 × 10^−6^ K^−1^)^[^ ^26^ ^]^ is very similar to the ones of HfO_2_ and HZO and significantly higher than the one from W (4.5 × 10^−6^ K^−1^).^[^ ^19^ ^]^

In this context, the use of W electrodes to promote the formation of the *o‐*phase with improved ferroelectric performance is investigated in this study. We first grew W/ZrO_2_/W structures onto Si/SiO*_x_* substrates by ion beam sputtering at a maximum temperature of 330 °C. We then performed the crystallization of the ZrO_2_ layer by nanosecond laser annealing (NLA). This was done by implementing a simulation‐guided approach to determine the appropriate NLA conditions. It was previously reported the successful use of the NLA process to promote the fabrication of Si‐doped HfO_2_ and HZO capacitors,^[^ ^27^, ^28^, ^29^ ^]^ but the use of NLA to promote the crystallization of the ZrO_2_ films has not previously been investigated. As well as being advantageous for producing rapid crystallization, compared to standard annealing NLA can strongly reduce W oxidation as the annealing time is very short, in the ns range. Overall, we provide experimental evidence for the formation of the *o‐*phase in the ZrO_2_ films in the Si/SiO_x_/W/ZrO_2_ film stack and we demonstrate stable ferroelectric performance.

## 2 Experimental Section

### 2.1 Device Fabrication and Characterization

W(14 nm)/ZrO_2_(8 nm)/W(22 nm) structures were grown by ion‐beam sputter deposition (IBSD) onto p‐type (100) Si/SiO*_x_* substrates (Si‐Mat). The IBSD chamber is equipped with a multitarget carousel system that allows the deposition of the bottom W and ZrO_2_ layers without breaking the vacuum. The vacuum chamber was first evacuated down to a low pressure of 1 × 10^−6^ mbar prior to the deposition. During the deposition, the substrate was kept at a temperature of 22 °C and 330 °C for W and ZrO_2_, respectively, at a distance of 87.3 mm from the target. The gas pressure inside the chamber was maintained constant at 2.5 × 10^−4^ mbar. A gas flow of 8.0 ml min^−1^ of Ar was introduced into the ion beam gun and the atoms were ionized in the ion source with an RF‐power of 100 W. The ion beam was further accelerated at 500 V and 600 V for W and ZrO_2_, respectively, and the ion beam current was maintained at 13 and 14 mA, respectively. Then, for the top W electrode deposition a shadow mask was used to pattern circular electrodes, with 0.8 mm diameter. After the deposition of the structures, nanosecond laser annealing was performed in air, at room temperature, using a KrF laser (*λ* = 248 nm). The laser, with an energy density of ≈0.4 J cm^−2^, was emitting light with a pulse length of 25 ns, and a repetition rate of 10 Hz, during 10 s (100 pulses). The spot size of the laser is 10 × 5 mm^2^, which is large enough when compared to the device size in order to have a uniform annealing.

The structural characterization of the deposited layers was performed using X‐ray diffraction (XRD), which was carried out in a Bruker D8 Advance DaVinci (Germany) diffractometer at room temperature over the 2*ē* range = 22–36°, in a Bragg‐Brentano configuration using CuK*α* radiation (*λ* = 1.5406 Å). The microstructural characterization of the sample was performed by high‐resolution transmission electron microscopy (HRTEM) using a JEM ARM200F instrument operated at 200 kV. The cross‐section TEM specimen was prepared for analysis by mechanical polishing down to ≈30 µm, followed by ion milling in a Gatan PIPS machine at 4 kV accelerating voltage and 7^o^ incidence angle. Low‐voltage ion milling was used as a final polishing stage in order to reduce the amorphous surface layer enveloping the specimen. Electron backscatter diffraction (EBSD) was performed using a ZEISS GeminiSEM 300 at accelerating voltages between 15 and 20 kV and a beam aperture between 60 and 120 µm. Electron backscatter patterns (EBSPs) were collected on an Oxford instruments Symmetry S3 CMOS‐based detector. Films were grounded for EBSD measurements using Ag paste. Ferroelectric hysteresis loops (*P‐E*) were measured at room temperature with a modified Sawyer‐Tower circuit using a triangular signal of 0.5, 1, and 2.5 kHz.

### 2.2 Computational Simulation

**Figure 1:**
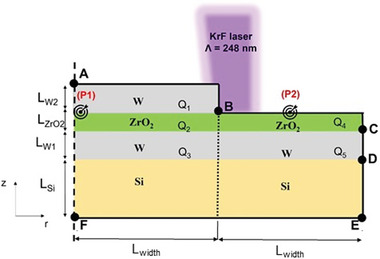
Simulated schematic view of boundary conditions of laser beam interaction with material layers. P1: ZrO_2_ surface under W electrode. P2, ZrO_2_ surface exposed to air.

**Figure** **1** shows the geometry of the W/ZrO_2_/W/Si(substrate) and the 2D axisymmetric domain used in the simulations of ns‐laser annealing multilayer stack. For simplicity, we neglected the existence of the native SiO*_x_* layer. The COMSOL Multiphysics software was used to perform the simulations of the laser heating and heat transport in the multilayer stack by solving the nonlinear inhomogeneous heat conduction equation,

(1) $$\rho \left(T\right)c\left(T\right)\frac{\partial T\left(r,z,t\right)}{\partial t}=\nabla \cdot \left[k\left(T\right)\vec{\nabla }T\left(r,z,t\right)\right]+Q\left(r,z,t\right)$$

where *T*(*r*,*z*,*t*) is the temperature, *ρ* is the density, *c* is the specific heat, *k* is the thermal conductivity of the material. The term *Q*(*r*,*z*,*t*) is the volumetric heat source due the absorption of incident laser radiation by the material. Using the Beer–Lambert law and assuming that the laser radiation intensity is uniformly distributed over the irradiated area, *Q*(*r*,*z*,*t*) can be expressed as:

(2) $$Q\;\left(r,z,t\right)=\alpha \left(T\right)\left(1-R\left(T\right)\right)q\left(t\right)exp\left(-\int_{0}^{z}\alpha \left(T\right)dz^{\prime }\right)$$

with *α* is the material absorption coefficient, *R* as its reflection coefficient, and *q*(*t*) is the laser pulse temporal profile, described by

(3) $$q\;\left(t\right)=\frac{W}{\tau }\sin^{2}\left(\frac{\pi t}{2\tau }\right)$$

where *W* represents the laser energy density and *τ* is the half amplitude of the pulse duration. The volumetric heating source terms *Q*_1_‐_5_ for the different layers in Figure 1 are written as:

(4) $$Q_{1}=\alpha_{W}q\left(t\right)\left(1-R_{W}\right)e^{-\alpha_{W}\times \left(L_{tot}-z\right)}$$

(5) $$Q_{2}=\alpha_{ZrO2}q\left(t\right)\left(1-R_{W}\right)e^{-\alpha_{W}\times L_{W2}}e^{-\alpha_{ZrO2}\times \left(L_{tot}-L_{W2}-z\right)}$$

(6) $$\begin{array}{c} Q_{3}=\alpha_{W}q\left(t\right)\left(1-R_{W}\right)e^{-\alpha_{W}\times L_{W2}}e^{-\alpha_{ZrO2}\times L_{ZrO2}}e^{-\alpha_{W2}\times \left(L_{tot}-L_{W2}-L_{ZrO2}-z\right)} \end{array}$$

(7) $$Q_{4}=\alpha_{ZrO2}q\left(t\right)\left(1-R_{ZrO2}\right)e^{-\alpha_{ZrO2}\times \left(L_{tot}-L_{W2}-z\right)}$$

(8) $$\begin{array}{ccc} Q_{5} & = & \alpha_{W}q\left(t\right)\left(1-R_{W}\right)\left(1-R_{ZrO2}\right)e^{-\alpha_{ZrO2}\times L_{ZrO2}}\\ & & \times \;e^{-\alpha_{W2}\times \left(L_{tot}-L_{W2}-L_{ZrO2}-z\right)} \end{array}$$

To solve Equation (1), suitable boundaries and initial conditions were defined. The initial temperature of the multilayer stack was set in the computational model to the room temperature:

(9) $$T\left(r,z,t=0\right)=T_{\mathrm{amb}\cdot }$$

where *T*_amb_ is the ambient temperature, 293,15 K. This fixed temperature assumption was also used for the Si substrate bottom surface $\left(\underline{EF}\right)$:

(10) $$T\left(r,z=0,t\right)=T_{\mathrm{amb}}$$

For $\underline{AF}$ surface, the axial symmetry boundary condition was considered:

(11) $$\frac{dT}{dr}=0$$

The boundary conditions for surfaces $\underline{AB}$, $\underline{BC},\;\underline{CD},\;\underline{DE}$, consider radiation heat losses to the ambient using the Stefan‐Boltzmann law:

(12) $$-n\cdot \left(k\vec{\nabla }T\right)=\sigma \varepsilon \left(T^{4}-T_{\mathrm{amb}}^{4}\right)$$

where *n* is the surface normal vector, *ε* is the surface emissivity, *σ* is the Stefan‐Boltzmann constant. We also assume a perfect thermal contact between the W/ZrO_2_/W/Si layers and neglect the interface thermal resistance effect.

The heat transfer module of COMSOL Multiphysics was utilized to solve the time‐dependent heat transport Equation (1) in a 2D axisymmetric domain (Figure 1) using the finite element method. A mapped mesh is used for domain discretization with a minimum mesh size of 1 nm along *z* and 25 nm along *r*. The simulations were performed for a single laser pulse, since there is no thermal effect of one pulse on the next pulse, due to a long time between laser pulses (pulse repetition frequency 10Hz). A transient analysis was thus done with time scale varying from 0 ns to 200 ns and variable time steps selected from 0.2 to 10 ns using the implicit backward differentiation formula (BDF) method. The resulting linear system of equations was solved using the PARDISO solver. The materials thermophysical properties were expressed as a function of temperature to reflect more realistic physical phenomena. Since the ns‐laser beam wavelength used is 248 nm, the materials optical properties corresponding to 248 nm were used for simulating the temperature distribution across the multilayer stack. The full list of parameters used in the simulations is given in Tables S1 and S2 of the Supporting Information.

## 3 Results and Discussion

**Figure 2:**
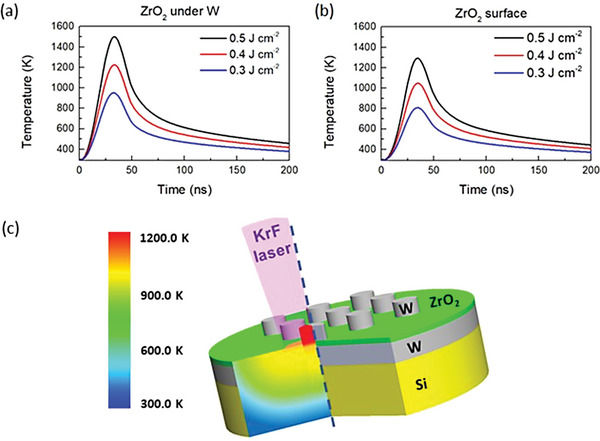
Simulated temperature distribution as a function of time for the NLA process with a fluence of 0.4 Jcm^−2^ for a) ZrO_2_ surface under the W top electrode (P1 in Figure 1) and b) uncovered ZrO_2_ surface (P2 in Figure 1). c) Schematic representation of the structure on the right side and in‐depth temperature gradient distribution across the layers. The dashed blue line separates both sides.

In order to determine the optimal laser fluence for the crystallization of ZrO_2_ in the as‐grown films, a knowledge of the temperature distribution and temporal evolution across the multilayer structure is essential.^[^ ^30^ ^]^ Thus, the heating and cooling rates need to be calculated during the process, and the right electrode materials used to give optimum heat conduction and generation of thermal strain in the oxide film necessary for stabilization of the ferroelectric phase. In this regard, W is a good choice because of its high thermal conductivity and the aforementioned TEC value. **Figure** **2**a,b shows the temperature profile as a function of time for different laser fluences on the ZrO_2_ surface under a top W electrode, compared to a ZrO_2_ surface with no electrode. It is observed that the temperature reached in the ZrO_2_ layer under the top W electrode (P1 in Figure 1) is higher than that in the ZrO_2_ surface exposed directly to the laser beam (P2 in Figure 1), irrespective of the laser fluence used. This is due to the higher heat absorption by the *W* layer upon laser incidence, when compared to the uncoated ZrO_2_, allowing it to reach higher temperatures. For the absorption length of *W* (see Table S2, Supporting Information), ≈98% of laser energy (not reflected) will be absorbed in the capping *W*, leading to a significative heating of the top W electrode. Due to the large absorption length for ZrO_2_ (≈128 nm) almost no laser energy is absorbed in the ZrO_2_ film. This means that only ≈2% of laser energy transmitted through the top electrode will reach the bottom *W* electrode. In the case of laser incidence directly in ZrO_2_ film, the bottom electrode gets heated, since almost all laser energy transmitted will reach it. Therefore, the lower temperature of the uncovered ZrO_2_ film upon laser incidence, compared to the W‐electrode‐covered‐ZrO_2_ layer is explained by the contribution of radiative heat losses, resulting in a lower net heat absorption in this layer.

Typically, the *o‐*phase in ZrO_2_ films is achieved after a rapid‐thermal annealing (RTA) at a temperature equal to or higher than 600 °C.^[^ ^30^, ^31^ ^]^ From our simulations was predicted that a minimum laser fluence of 0.4 J cm^−2^ should be used in the NLA process in order to crystallize the ZrO_2_ layer. Figure 2c shows on the right side of the blue dashed line a schematic representation of the structure, while the left side shows the simulated in‐depth temperature profile across the structure, 30 ns after laser incidence, for a NLA process with a laser fluence of 0.4 J cm^−2^. The largest temperature difference in Figure 2c occurs in the Si substrate, ≈600 K, while the temperature difference is only 100K in the ZrO_2_ thin film. The high temperatures, above 900 K, in the ZrO_2_ layer, not only allow the kinetic barrier for the tetra‐ortho phase transition to be overcome but also lead to the stabilization of the *o‐*phase due to the high tensile thermal stresses induced by the large difference in TEC of W and ZrO_2_ layers. The top W layer, thus plays a fundamental role in the NLA process of the ZrO_2_ film, working not only as a capping protection layer from laser energy pulse, but also contributing to the heating of ZrO_2_ layer and generating the tensile thermal stresses necessary for stabilization of the *o‐*phase.

**Figure 3:**
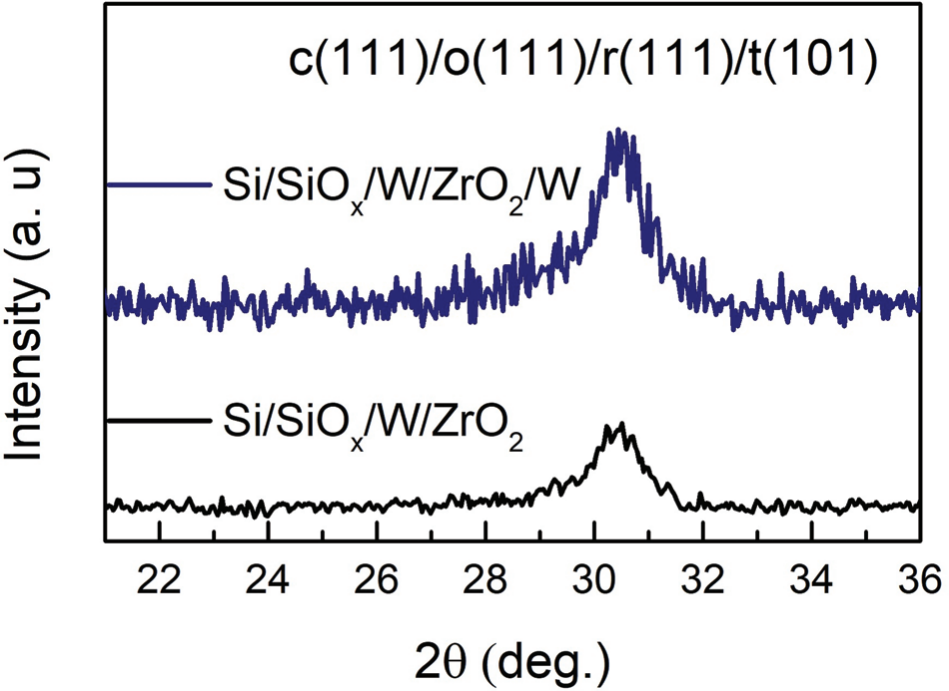
XRD pattern of the Si/SiOx/W/ZrO_2_ and Si/SiO*x*/W/ZrO_2_/W structures.

After performing the NLA with a fluence of 0.4 J cm^−2^, the samples were structurally characterized by X‐ray diffraction (XRD). **Figure** **3** shows the XRD pattern for the Si/SiO*_x_*/W/ZrO_2_ and Si/SiO*_x_*/W/ZrO_2_/W structures after the NLA. One significant diffraction peak centered at 2*θ* = 30.45° (*d* = 2.93 Å) is observed in both structures. No other peaks were observed, indicating no significant WO*_x_* formation.

The 30.45° diffraction peak could be attributed to the presence of the cubic (*c*)‐phase (111), *o*‐phase (111), tetragonal (*t*)‐phase (101), or the *r*‐phase (111) planes.^[^ ^24^, ^32^ ^]^ The *c‐*phase and *t‐*phase nanoscale ZrO_2_ are known for their paraelectric and antiferroelectric characteristics, respectively.^[^ ^33^ ^]^ Owing to the limitation of the XRD technique, it is not possible to unequivocally distinguish between the ferroelectric *o‐*phase or *r‐*phases and other nonferroelectric phases. Moreover, the XRD patterns reveal that no significant differences are observed in the XRD peak when comparing both samples. In addition, the samples were amorphous before any NLA process.

**Figure 4:**
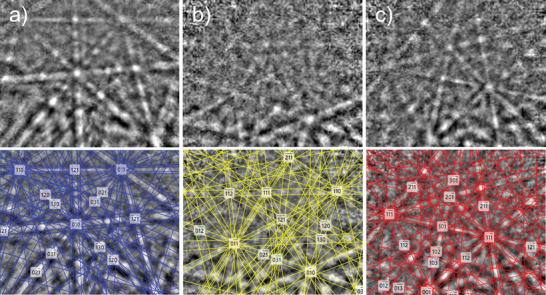
Example of electron backscatter diffraction patterns (top) and corresponding fits (bottom) for a) orthorhombic phase fit with 10 bands and MAD of 0.60°, b) monoclinic phase fit with 9 bands and MAD of 0.68°, and c) tetragonal phase fit with 9 bands and MAD of 0.58°.

We also used electron backscatter diffraction (EBSD) and transmission electron microscopy to clearly identify the phases present in the ZrO_2_ films without and with the top W layer, respectively. In the case of EBSD measurements beneath the W layer is not possible. For EBSD, 15,000 election backscatter patterns were collected across an ≈1 mm area of the film. Electron backscatter diffraction patterns (EBSPs) were indexed using 12 bands and a Hough resolution of 70. A polycrystalline structure was identified, though the low‐resolution of EBSPs collected prevented indexing for ≈75% of patterns. The small film thickness precluded collection of high‐resolution EBSP despite exposure times of up to 500 ms. Additionally, it is not possible to index patterns where grains were smaller than the electron interaction volume (<10 nm). Another consideration, given the low pattern resolution, is the effect of pseudosymmetry and mis‐indexing of orthorhombic and tetragonal phases. For low‐resolution patterns, it can be challenging to distinguish between some orientations of *o‐*phase and *t‐*phase. Despite the aforementioned challenges, the presence of *o‐*, *m‐*, and *t‐* phases was confirmed from higher‐quality patterns, for example, those in **Figure** **4** (a,b, and c for *o‐*, *m‐*, and *t‐* phases respectively). For these three EBSPs, 9 bands were matched for the *t‐* and *m‐*phase patterns, and 10 bands for the *o‐*phase. The indexed patterns have a mean angular deviation (MAD) of 0.60°, 0.68°, and 0.58° respectively for *o‐*, *m‐*, and *t‐* phases. Mean angular deviation (MAD) is defined as the angular deviation between the observed and simulated lattice plane orientations. A value below 1° is typically acceptable for phase identification, but in the case of pseudo‐symmetric phases, it has been found that MAD values <0.7° significantly reduce the rate of mis‐indexing.^[^ ^34^ ^]^

Given the high number of nonindexed patterns and the potential for mis‐indexing, an absolution phase ratio cannot be obtained. However, of the patterns indexed the most common fitting is the *o‐*phase (≈65%) and the *o‐* patterns have the average mean MAD value (0.9°) which supports the identification of *o‐* as the mostly likely primary phase. Additionally, of the *o‐* indexed regions, a texturing is demonstrated, with the [111] neat surface normal (See Figure S1, Supporting Information).

**Figure 5:**
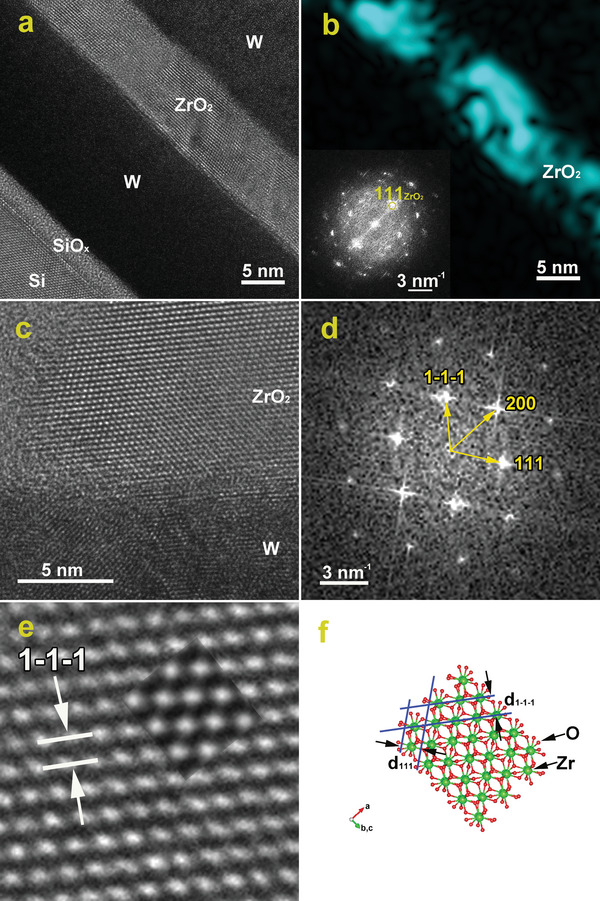
a) Large‐area HRTEM image of the Si/SiO*_x_*/W/ZrO_2_/W structure; b) Spatial distribution of the (111)_ZrO2_ lattice fringes obtained by filtering the power spectrum (inserted) of the HRTEM micrograph in (a); c) HRTEM image of a zone axis oriented ZrO_2_ grain at the W/ZrO_2_ interface. d) FFT pattern from an area containing the ZrO_2_ crystallite in (c); e) enlarged HRTEM image of the ZrO_2_ crystallite in (c) and the embedded simulated HRTEM pattern of the orthorhombic ZrO_2_ (defocus ‐10 nm, thickness 40 nm); f) Atomic structural model of ZrO_2_ in the [01‐1] orientation in perfect agreement with structural data provided by the experimental images.

**Figure** **5**a shows a large‐area HRTEM image exhibiting the W/ZrO_2_/W layers deposited on top of the Si substrate with a native SiO*_x_* layer of 2–3 nm. The thickness of the ZrO_2_ layer is 8 ± 1 nm, while the bottom W layer thickness is 14 ± 1 nm. Moreover, the interfaces between the substrate and the W layer and also between the W layers and the ZrO_2_ layer are clean with no visible discontinuity.

The fully crystallized state and texturing of the ZrO_2_ layer can be observed on the large‐area HRTEM image showing (111)_ZrO2_ lattice fringes with a preferential orientation parallel to the substrate. The film texturing is better illustrated by the Fourier‐filtered image in Figure 5b. The (111)_ZrO2_ texturing of the ZrO_2_ thin film is demonstrated on one side by the power spectrum (inserted in Figure 5b), showing the strong (111)_ZrO2_ spots aligned along a direction perpendicular to the substrate. On the other hand, we mapped the spatial distribution of the (111)_ZrO2_ lattice fringes in the HRTEM micrograph by selecting the (111)_ZrO2_ peak in the associated whole‐frame power spectrum. The filtered HRTEM image shows that the (111)_ZrO2_ lattice fringes are visible and aligned (approximately) parallel to the substrate across the whole HRTEM micrograph, thus validating the ZrO_2_ film texturing.

In order to analyze the crystalline phase in the ZrO_2_ film, we examined the Fast Fourier Transform (FFT) of the HRTEM image of a zone axis‐oriented ZrO_2_ grain selected from the thinner part of the specimen (Figure 5c,d). The *o‐*phase can be reliably discerned from the monoclinic phase by analyzing the {111} diffraction peaks: the (111)o peak corresponds to an interplanar distance of 0.3 nm between the equivalent {111} planes, while in the case of the monoclinic phase the (111)m peak splits in two, corresponding to the {111}m and {11‐1}m families of planes, spaced at 0.286 nm and 0.322 nm, respectively. In our case, from the measurements performed both on the HRTEM micrograph and on the FFT picture performed on an area which contains an oriented ZrO_2_ crystallite (Figure 5b), we obtained the following interplanar distances: 0.296 nm + −0.004 nm, which can be assigned to {111} planes of the *o*‐phase with space group Pca_21_,^[^ ^35^ ^]^ but this measured distance can be assigned also to the {101} family of planes of the tetragonal phase with space group P4_2_/nmc. The (101) planes of the tetragonal phase are spaced at a 0.3006 nm distance, which is within the error limits of our measurements. Since the FFT pattern, shown in Figure 5d, contains a well‐defined pattern of spots, thus proving a high crystallization degree in terms of grain size and preferential crystallographic orientation, we measured and assigned three peaks to the (1‐1‐1), (111), and (200) planes of the *o‐*structure of ZrO_2_ the crystallite being viewed along the [01‐1] zone axis. The structure parameters for this spatial group are: *a* = 0.52336 nm, *b* = 0.52684 nm, and *c* = 0.54184 nm. Figure 5d shows a magnified view of a ZrO_2_ crystallite, which contains the atomic structural model of the orthorhombic structure embedded to emphasize the correct interpretation of the experimental results.

The HRTEM measurements performed on the ZrO_2_ crystallite below the top W layer confirm the textured growth with a preferential orientation of the {111} family of planes. For the direction along the normal surface, bearing in mind the indexation used in the FFT pattern from Figure 5b, the [1‐1‐1] direction makes an angle of ≈5^o^ with the surface normal, whereas, for the other direction, in‐plane, the [111] direction makes an angle of ≈12^o^ with the direction perpendicular to the normal surface, as presented in Figure S3, Supporting Information. Therefore, the ZrO_2_ is textured with the [111] direction almost parallel to the surface normal, similar to what was observed from EBSD results performed on the ZrO_2_ film without the W top layer.

In Figure 5f, we simulate using VESTA software the atomic structural model of ZrO_2_ along the [01‐1] zone axis and we also determined the distances between the (1‐1‐1) and (111) set of planes. It can be observed that there is a perfect agreement between the simulated structure and the experimental data provided by the HRTEM pictures. In order to be able to confirm the crystalline structure of the ZrO_2_ crystallite, we simulated HRTEM patterns and atomic structural models, using the multislice technique, with the use of the *x*HREM software kit. In each case, a series of simulated images were generated according to the sample thickness and the defocus of the objective lens, as it is displayed in Figure S4, Supporting Information. The intensity modulation of HRTEM images was investigated and associated with the atomic structure by simulating the HRTEM patterns with the multislice technique. A matrix of HRTEM patterns was simulated along the zone axis in which the ZrO_2_ crystallite is oriented: B = [01‐1] for the orthorhombic structure of ZrO_2_. Moreover, in order to confirm the correct interpretation of the experimental data, the HRTEM simulated image was overlapped with the experimental HRTEM image, as shown in Figure 5e. It is possible to observe a good agreement between the experimental pattern and the one simulated for a defocus of −10 nm and a thickness of 40 nm and therefore we were able to further confirm the presence of the *o‐*phase in the ZrO_2_ film.

**Figure 6. a):**
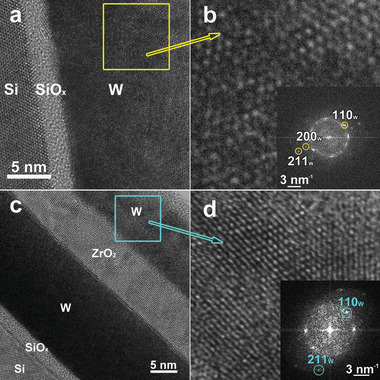
HRTEM image of the Si/SiO*_x_*/W structure and b) magnified image of the lattice fringes of W structure and the FFT pattern embedded in the HRTEM picture, performed on an area marked by the yellow square in (a), in which we indexed and assigned two peaks for the cubic structure of the bottom W structure, c) HRTEM image of the Si/SiO*_x_*/W/ZrO_2_/W structure and d) magnified image of the lattice fringes of W structure together with the FFT pattern of the area marked by the blue square in (c).

The HRTEM images in **Figure** **6** refer to the microstructural characterization of the W bottom and top layers. Due to the low sputtering rate of W during the Ar+ ion milling and the shadowing effect played by the hard W layers, we could not identify a location where both layers were simultaneously thin enough for HRTEM imaging. Therefore, the HRTEM analyses of the top and bottom W layers were performed in two different locations where either the top or the bottom W layer had the right thickness for HRTEM imaging. Figure 6a,b,c,d are large‐area and enlarged HRTEM images of the bottom and top W layers, respectively. The enlarged HRTEM images in Figure 6b,d contain the FFT pattern performed on the square areas marked in Figure 6a,c. By analyzing the FFT patterns we could identify the cubic crystalline structure of metallic W (space group Im‐3m, *a* = 0.31648 nm) as a unique phase inside both the bottom and top layers. At this level, by using this technique, no WO*_x_* structure could be identified, thus confirming the XRD results where no trace of WO*_x_* phase is identified.

We observed that with a fluence of 0.5Jcm^−2^, the surface of the ZrO_2_ layer (without a top W layer) became damaged, as shown in the Figure S2, Supporting Information. However, with a fluence of 0.4 J cm^−2^ we do not observe a significant damage of the layers, as can be seen in the HRTEM images. In addition, the temperature difference predicted by the simulation in the ZrO_2_ layer, as discussed before, shouldn´t have any significant effect on the uniformity of the ZrO_2_ crystallization process, as can be observed in the HRTEM analysis from Figure 5.

**Figure 7. a):**
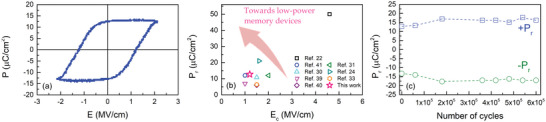
Polarization (*P*)‐electric field (*E*) hysteresis loop for the W/ZrO_2_/W film capacitor. b) Remanent polarization, *P_r_*, as a function of the coercive field, *E_c_* for different capacitors based on ZrO_2_ thin films. The arrows show the aimed direction for device improvement. c) *P_r_* as a function of the number of field switching cycles for W/ZrO_2_/W thin film capacitor.

**Figure** **7**a shows the room temperature pristine polarization‐electric field (P–E) hysteresis loops of the W/ZrO_2_/W film capacitor. A well‐saturated hysteresis loop was obtained, which confirms the ferroelectric nature of the ZrO_2_ film. To further confirm the ferroelectric nature of the films, frequency‐dependent P‐E hysteresis loops are shown in Figure S5, Supporting Information. With increasing frequency, there is a slight decrease of *P_r_* and *P_s_*, while *E_c_* increases. A similar behavior was observed in HZO films.^[^ ^36^ ^]^ The average values of *P_s_*, *P_r,_* and *E_c_* for the W/ZrO_2_/W film capacitor are 12.8 µC cm^−2^, 12.7 µC cm^−2^ and 1.2 MV cm^−1^, respectively. ZrO_2_ films annealed by rapid thermal annealing (RTA) reveal the formation of a WO_3_ layer with a 1–2 nm thickness. This layer forms when W electrodes scavenge oxygen during annealing, leaving the ZrO_2_ oxygen deficient.^[^ ^37^ ^]^ The existence of this WO_3_ layer leads to the observation of a pronounced imprint effect. In addition, the NLA sample revealed a lower *E_c_* and a higher *P_r_*, which are both crucial for low‐power memory applications. **Table** **1** shows a comparison between the average values of *P_s_*, *P_r,_* and *E_c_* for the W/ZrO_2_/W film capacitor obtained in this work with the ones presented in the recent literature based on ZrO_2_ thin films capacitors. The high *P_r_* and *P_s_* values together with low *E_c_* confirm that the present capacitors are promising for future nonvolatile memory. As observed from Table 1, there are no reports on sub‐10 nm thick ferroelectric ZrO_2_ films with *P_r_* higher than 10 µC cm^−2^ and a *E_c_* below 1.5 MV cm^−1^. In order to further elucidate this, we have plotted the *P_r_* as a function of *E_c_*, Figure 7b. With the aim of achieving low‐power memory devices, it is possible to observe that there is only a previous report of a film with a similar *P_r_* but with lower *E_c_*, than the values reported in this work, and the film was 12.5 times thicker than in this work. Therefore, the present capacitors exhibit a high *P_r_* and a low *E_c_*, which is crucial for memory devices.

**Table 1:** Comparison of the thickness, *P_s_*, *P_r,_* and *E_c_* values for different capacitors based on ZrO_2_ thin films

Structure	Thickness (nm)	*P_r_* [µC cm^−2^]	*P_s_* [µC cm^−2^]	*E_c_* [MV cm^−1^]	Ref.
Pt/ZrO_2_/Pt	6.5	≈50	≈57	4.6	[22]
Nb:STO/ZrO_2_/Au	8	10.8	10.8	1.5	[30]
Si/SiO_2_/ZrO_2_/TiN	10	≈13	≈22	‐	[38]
TiN/ZrO_2_/TiN	45	7	≈10	≈1	[39]
Ge/ZrO_2_/Al	17	6	≈13	1.5	[40]
Pt/ZrO_2_/Pt	100	≈12	≈22	≈1	[41]
Pt/ZrO_2_/Pt	19.6	≈12	≈12	≈2.0	[31]
Pt/ZrO_2_/Au	5	21	44	1.6	[24]
Pt/ZrO_2_/Pt	7.5	≈6.4	≈6.4	≈1.5	[33]
W/ZrO_2_/W	8	12.7	12.8	1.2	This work

Figure 7c shows the evolution of both +*P_r_* and –*P_r_* as a function of the number of cycles. It is possible to observe a slight increase of the |+*P_r_|* and |–*P_r_|* from ≈12.7 µC cm^−2^ up to ≈17 µC cm^−2^ after 1.8 × 10^5^ cycles. This increase in Pr of 4.3 µC cm^−2^ is attributed to the so‐called wake‐up effect. This wake‐up effect is ascribed to the migration of point defects (mainly oxygen vacancies) and/or a transition from nonferroelectric (*m‐* and *t‐*phase) to the ferroelectric phase (especially at the interfacial region).^[2]^ After this, both +*P_r_* and *–P_r_* values remain stable up to 6.0 × 10^5^ cycles, confirming the good endurance of the devices.

## 4 Conclusions

In this work, we have demonstrated the effectiveness of nanosecond laser annealing of amorphous ZrO_2_ films with W electrodes, for creating crystalline ferroelectric ZrO_2_ films. The optimum conditions of the rapid laser annealing were guided by COMSOL Multiphysics simulations of laser heating of the multilayer stack. Using XRD, EBSD, and TEM analyses, the formation of an orthorhombic phase of ZrO_2_ was confirmed as the ferroelectric phase in the films. The films showed a spontaneous polarization of ≈12.8 µC cm^−2^, a remnant polarization of 12.7 µC cm^−2^, a coercive field of 1.2 MV cm^−1^, and a polarization retention of 6.0 × 10^5^ cycles. A small wake‐up effect was also observed, attributed to the migration of point defects such as oxygen vacancies, and/or a transition from nonferroelectric (*m‐* and *t‐*phase) to the ferroelectric *o*‐phase. Overall, this study shows the strong potential for the fabrication of ferroelectric ZrO_2_ thin films by a nanosecond laser annealing process from amorphous precursor films. Such films have strong promise for the next generation of memory and sensing devices.

## Conflict of Interest

The authors declare no conflict of interest.

## Supporting information

Supporting Information

## Data Availability

The data that support the findings of this study are available from the corresponding author upon reasonable request.
