# Supplementary material for: Ferroelectric Orthorhombic ZrO2 Thin Films Achieved Through Nanosecond Laser Annealing
Source: Adv Sci (Weinh). 2023 Mar 22;10(15):2207390. doi: 10.1002/advs.202207390 (PMC10214231; doi:10.1002/advs.202207390)
Supplement: Supplementary file 1 — Supporting Information [file ADVS-10-2207390-s001.pdf]

ADVANCED SCIENCE
Open Access

Supporting Information

for *Adv. Sci.*, DOI 10.1002/advs.202207390

Ferroelectric Orthorhombic ZrO_2 Thin Films Achieved Through Nanosecond Laser Annealing

Anna P. S. Crema, Marian C. Istrate, Alexandre Silva, Veniero Lenzi, Leonardo Domingues, Megan O. Hill, Valentin S. Teodorescu, Corneliu Ghica, Maria J. M. Gomes, Mario Pereira, Luís Marques, Judith L. MacManus-Driscoll and José P. B. Silva**

Ferroelectric orthorhombic ZrO_2 thin films achieved through nanosecond laser annealing

Anna P. S. Crema[a,b,1], Marian C. Istrate[c,d,1], Alexandre Silva [a,b], V. Lenzi[a,b], Leonardo Domingues[a,b], Megan O. Hill[e], V. S. Teodorescu[c,d], Corneliu Ghica[d], Maria J. M. Gomes[a,b], M. Pereira[a,b], Luís Marques[a,b], Judith L. MacManus-Driscoll[e]*, José P. B. Silva[a,b,]*

[a]Physics Center of Minho and Porto Universities (CF-UM-UP), University of Minho, Campus de Gualtar, 4710-057 Braga, Portugal
[b]Laboratory of Physics for Materials and Emergent Technologies, LapMET, University of Minho, 4710-057 Braga, Portugal
[c]University of Bucharest, Faculty of Physics, Atomistilor 405, Magurele Ilfov 077125, Romania
[d]National Institute of Materials Physics, 105 bis Atomistilor, 077125 Magurele, Romania
[e]Dept. of Materials Science and Metallurgy, University of Cambridge, 27 Charles Babbage Rd., Cambridge, CB3 OFS, U.K.

[1] These authors contributed equally to this work.
* Corresponding authors' e-mails: josesilva@fisica.uminho.pt and jld35@cam.ac.uk

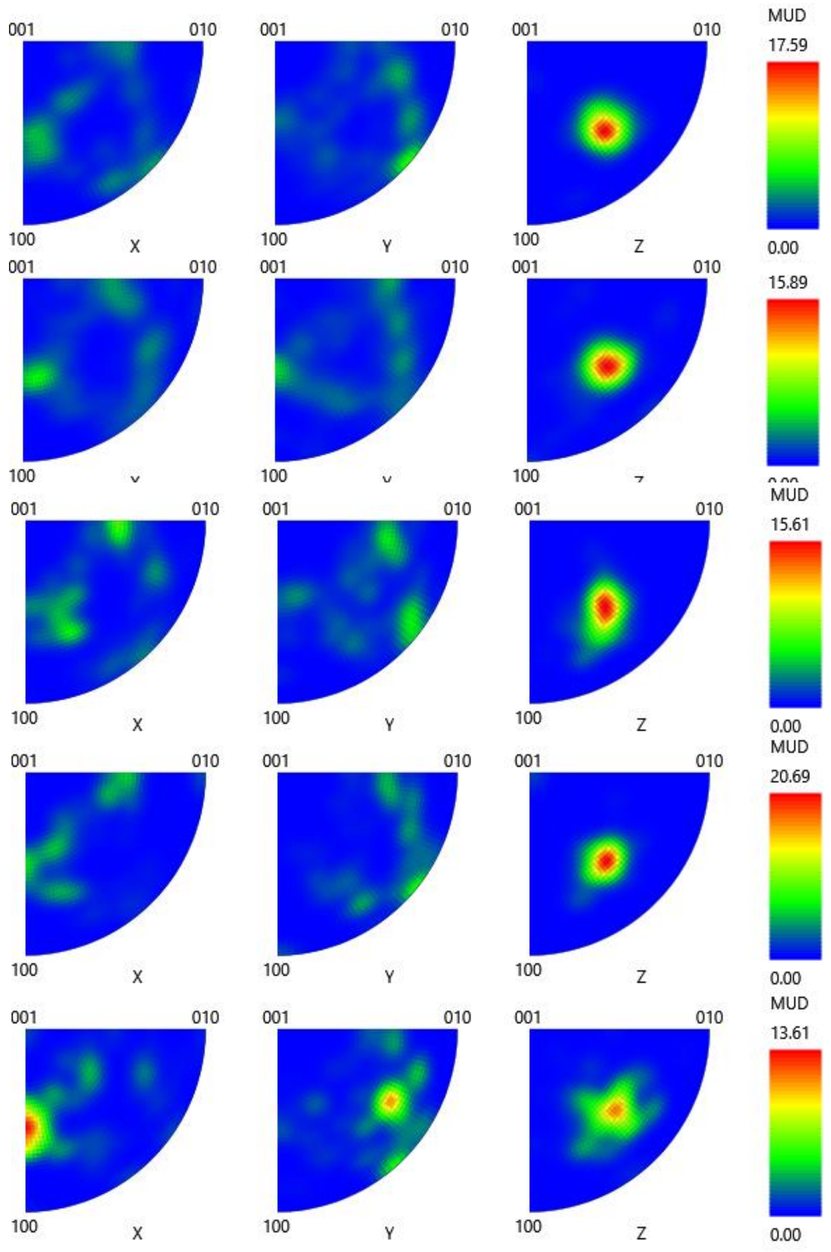

Figure S1. Contour inverse pole figures showing the strength of pole clustering along each axis, with Z being normal to the film surface. These are shown for patterns collected across 5 regions of the film. This is in terms of multiples of uniform density (MUD), comparing the clustering strength to that of a random distribution. Of the indexed orthorhombic patterns, the patterns appear to be highly textured with the film normal aligning closes to the [111] direction. However, more variation is observed for in-plane orientation.

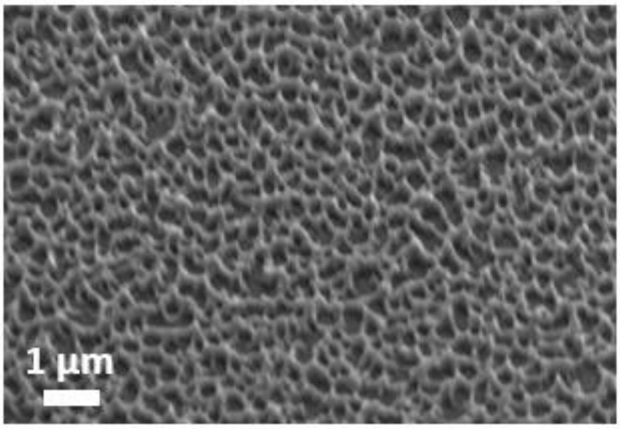

Figure S2. SEM image of the ZrO_2 layer after NLA process with a fluence of 0.5 Jcm^{-2}.

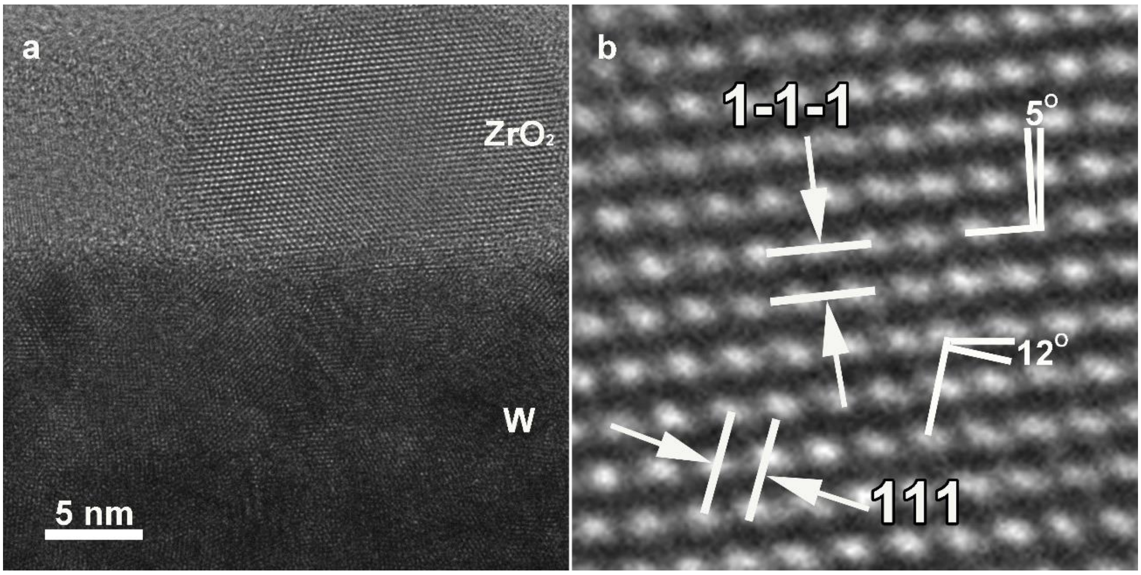

Figure S3. (a) HRTEM image of the ZrO_2/W multi-structure, (b) ZrO_2 crystallites showed in detail extracted from image (a) in which two angles were measured, [1-1-1] has an angle of ~5° to the surface normal, and [111] has an angle of ~12° with the direction perpendicular to the normal surface.

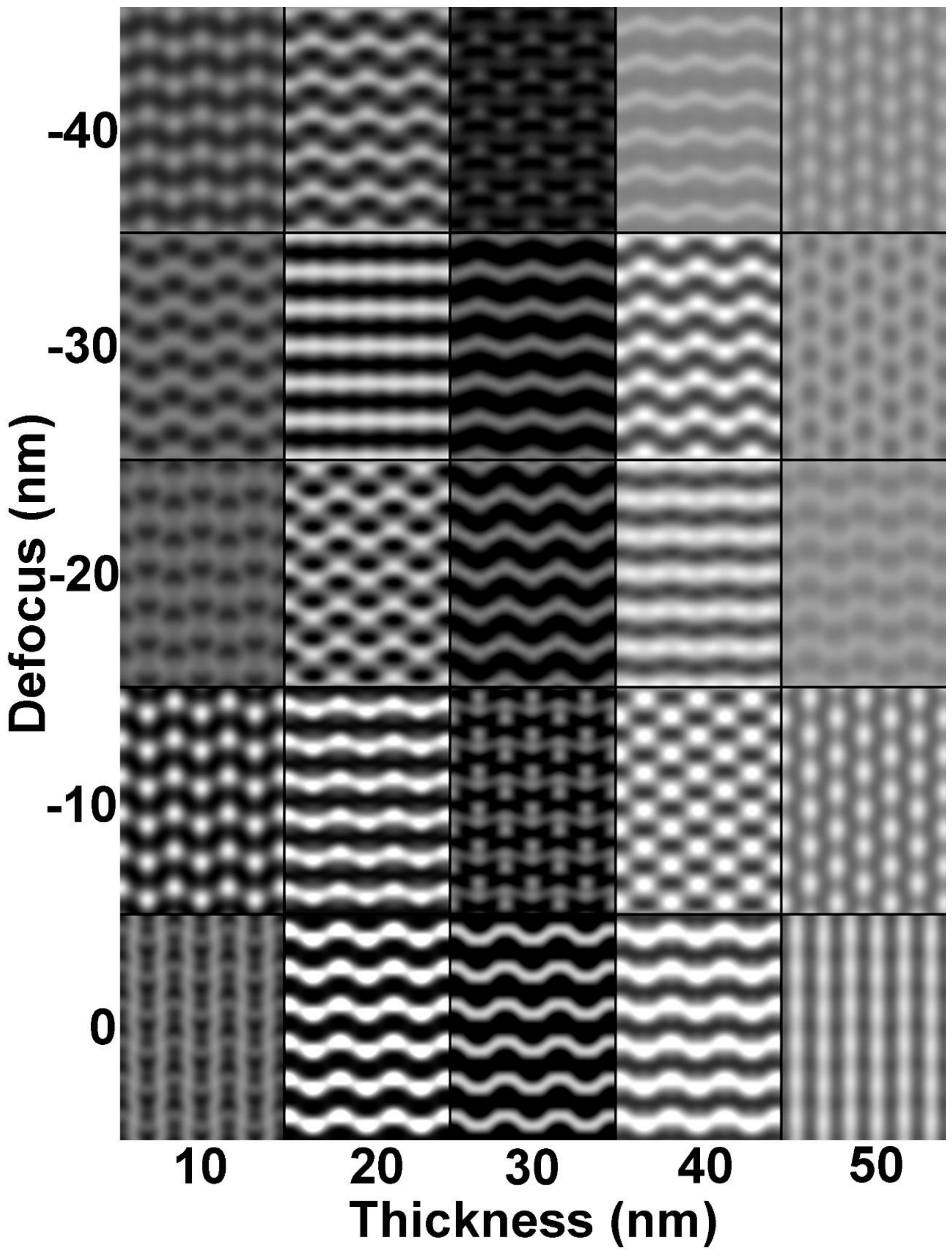

Figure S4. (a) HRTEM pattern simulation fit for orthorhombic ZrO_2 structure along B=[01-1] zone axis. The perfect fit between the simulated HRTEM images and the experimental one it is obtained at a defocus of -10 nm and a thickness of 40 nm as it is shown in Fig. 5(f).

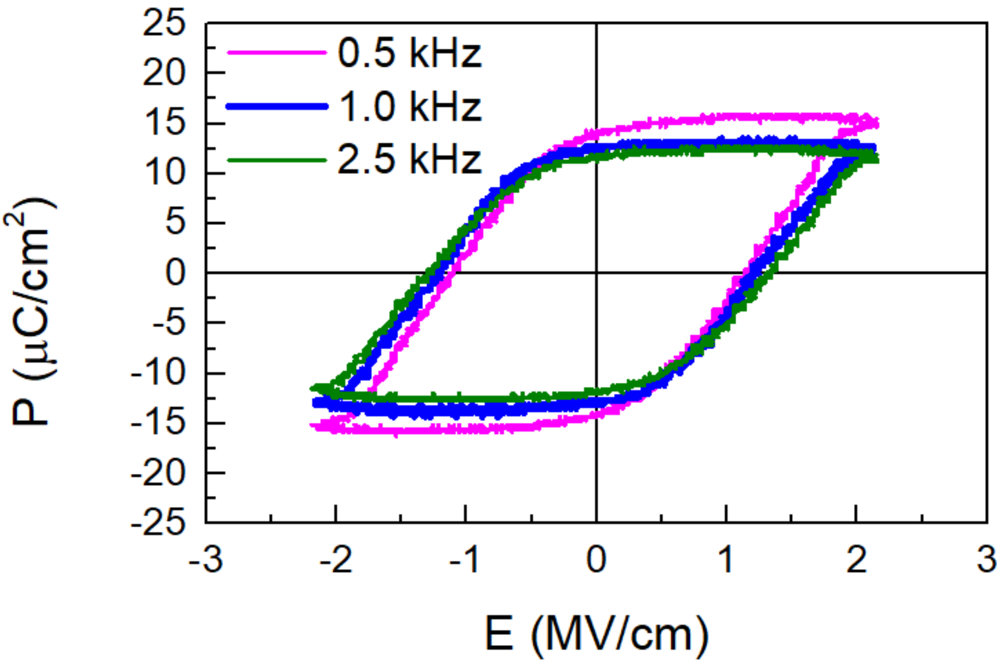

Figure S5. Frequency dependent polarization (*P*)-electric field (*E*) hysteresis loop for the $W/ZrO_2/W$ film capacitor.

Table S1. Laser parameters and simulation domain dimensions

Name	Description	Value	Unit
tau	Laser pulse	$2.5E^{-8}$	s
W	Laser Fluence	0.4	J/cm^2
λ	Wavelenght	$2.48\ E^{-7}$	m
L_{Si}	Tickness Silicon substrate	$5.0\ E^{-6}$	m
L_{W1}	Tickness bottom electrode	$1.6\ E^{-8}$	m
L_{W2}	Tickness top electrode	$2.2\ E^{-8}$	m
L_{ZrO2}	Tickness Zirconia layer	$8.0\ E^{-9}$	m
L_{width}	Eletrode radius	$0.4\ E^{-3}$	m

.

Table S2. Materials parameters used in simulation.

	Name	Description	value	Unit	Ref
	k	Thermal conductivity	k(T)	W/(m K)	1
	c	Heat capacity	c(T)	J/(kg K)	2
**Zirconia**	*ρ*	Density	*ρ* (T)	Kg/m^3	3
	α	Absorption coefficient*	7.82E6	m^{-1}	4
	R	Reflectance*	0.16	-	4
	ε	Surface emissivity	0.7	-	5
	k	Thermal conductivity	k(T)	W/(m K)	6
	c	Heat capacity	c(T)	J/(kg K)	7
**Tungsten**	*ρ*	Densitiy	*ρ* (T)	Kg/m^3	8,9
	α	Absorption coefficient*	1.68E8	m^{-1}	10
	R	Refletance*	0.64		10
	ε	Surface emissivity	*ε*(T)		11,12
	k	Thermal conductivity	k(T)	W/(m K)	6
**Silicon**	*c*	Heat capacity	c(T)	J/(kg K)	13
	ρ	Densitiy	*ρ* (T)	Kg/m^3	14
	ε	Surface emissivity	0.71	-	15

* Values for a wavelength λ = 248 nm.

Reference

1. W.D. Kingery, J. Francl, R.L. Coble and T. Vasilos, Journal of the American Ceramic Society, 37, 107, (1954).
2. L.B. Pankratz, Thermodynamic Properties of Elements and Oxides, US Bureau of Mines Bulletin 672 ,(1982).
3. D. Taylor, Trans. and J. of the Brit. Cer. Soc. 83(2), 32 (1984).
4. J. Desforges; L. Robichaud; S. Gauvin, Adv. in Mat. Sci.and Eng. 8285230 (2017).
5. H. Tanaka, S. Sawai, K. Morimoto; K. Hisano, J. of Thermal Anal. and Cal. (64), 867 (2001).
6. C.Y. Ho, R.W. Pow.and P.E. Liley, J. Phys. Chem. 1(2) 279, 1972.

7. G.K. White; S.J. Collocott, Journal of Physical and Chemical Reference Data, 13(4), p1251 (1984).
8. K. Wang; R. R. Reeber, Mat. Sci. and Eng. 23(3), 101-137 (1998).
9. G.K. White; R.B. Roberts. Hig.Temp.Hig. Press. 15, 321 (1983).
10. W. S. M. Werner; K. Glantschnig; C. Ambrosch-Draxl. J. Phys Chem Ref. 1013-1092 (2009).
11. W.E. Forsythe; E.M. Watson; J. Opt. Society America; 24, 114 (1934).
12. W.E. Forsythe; E.Q. Adams; J. of the Opt. Soc.of America, 35, 108 (1945).
13. P.D. Desai; J. of Phys. and Chem. Ref. Data, 15(3), p967 (1986).
14. C.A. Swenson. J. of Phys. and Chem. Ref. Data, 12(2), p179 (1983).
15. P. J. Timans. J. of App. Phys. 74, 6353 (1993).
